# The gut microbiome and pathology of Alzheimer’s disease

**DOI:** 10.1002/alz.090732

**Published:** 2025-01-09

**Authors:** Sithara Udeshini Dissanayaka, Stephanie R Rainey‐Smith, Ralph N Martins, Warnakulasuriya Mary Ann Dipika Binosha Fernando

**Affiliations:** ^1^ Centre of Excellence for Alzheimer’s Disease Research and Care, School of Medical and Health Sciences, Edith Cowan University, Joondalup, Western Australia Australia; ^2^ Murdoch University, Murdoch, Western Australia Australia; ^3^ Edith Cowan University, Perth, Western Australia Australia; ^4^ Alzheimer’s Research Australia, Perth, Western Australia Australia; ^5^ Macquarie University, Sydney, NSW Australia

## Abstract

**Background:**

Current literature focuses on the association between gut dysbiosis and the aggregation of Aβ, the development of tau protein, as well as neuroinflammation and oxidative stress associated with AD. Since the brain and gut are connected (gut‐brain axis), gut microbiota and their metabolites may influence AD progression, or vice versa, if AD pathogenesis impacts the microbiome. Observational studies have shown an altered taxonomic composition of gut microbiota in AD patients compared to cognitively normal (CN) controls.

**Method:**

This perspective study will explore taxonomic composition and gut microbial function (using metagenomic analysis) compared with Aβ aggregation, deposition of tau, and inflammatory biomarkers in cognitively normal individuals and preclinical AD individuals. We will also address the effect of dietary intake on gut dysbiosis to assess the effectiveness of healthy diet patterns as preventive approaches in AD.

**Results:**

The faecal samples of people with AD have been shown to have decreased microbial diversity and compositionally distinct microbiota. At the same time, correlations were found between gut microbiome differences and cerebrospinal fluid (CSF) tau levels, and plasma β‐amyloid (Aβ) biomarkers levels early in the progression of the disease. However, research into the gut and brain concerning AD progression and AD pathology remains very inconsistent with the reported data. Therefore, evaluation of the potential role of microbiota taxonomy in stool in the early detection of preclinical AD may lead to the development of novel directions in the investigation of the potential role of the gut in AD progression.

**Conclusion:**

The altered gut microbiota can influence increased Aβ deposits, tau phosphorylation, and neuroinflammation. Therefore, it is necessary to gain a better understanding of microbial community diversity at the species level and the function of these communities due to AD pathology as well as the effect of confounding factors like dietary intake.

